# Old-age mental telehealth services at primary healthcare centers in low- resource areas in Greece: design, iterative development and single-site pilot study findings

**DOI:** 10.1186/s12913-023-09583-5

**Published:** 2023-06-13

**Authors:** Antonios Politis, Theofanis Vorvolakos, Evaggelia Kontogianni, Maria Alexaki, Eleni-Zacharoula (Eliza) Georgiou, Eleutheria Aggeletaki, Maria Gkampra, Maria Delatola, Antonis Delatolas, Apostolos Efkarpidis, Elissavet Thanopoulou, Konstantinos Kostoulas, Vassiliki Naziri, Anna Petrou, Kalliopi Savvopoulou, Kostas Siarkos, Rigas Filippos Soldatos, Vasileios Stamos, Kim-Huong Nguyen, Iracema Leroi, Dimitrios Kiosses, Konstantinos Tsimpanis, Panagiotis Alexopoulos

**Affiliations:** 1 Department of Psychiatry, Eginition Hospital, National and Kapodistrian University of Athens, Vasilissis Sophias 72, Athens, 11528 Greece; 2 Division of Geriatric Psychiatry and Neuropsychiatry, Department of Psychiatry, Johns Hopkins Medical School, 600 N. Wolfe Street Meyer Building, Baltimore, MD 21287 USA; 3 Department of Psychiatry, School of Health Sciences, University General Hospital of Alexandroupolis, Democritus University of Thrace, University Campus, Dragana, Alexandroupolis, 68100 Greece; 4 Primary Healthcare Center of Andros, Chora, Andros, 84500 Greece; 5 Mental Health Services, University General Hospital of Patras, Department of Medicine, School of Health Sciences, University of Patras, Rion, Patras, 26504 Greece; 6 Nursing Services Department, General Hospital of Syros “Vardakeio and Proio”, Geor. Papandreou 2, Ermoupolis, 84100 Greece; 7 Primary Healthcare Center of Xanthi, Andrea Dimitriou 1, Xanthi, 67133 Greece; 8 Primary Healthcare Center of Tinos, Mark. Krikeli 18, Tinos, 84200 Greece; 9 Primary Heathcare Center of Chalandritsa, Chalandritsa Achaea, 25008 Greece; 10 Primary Healthcare Center of Soufli, Soufli Evros, 68400 Greece; 11 Primary Healthcare Center of Erymanthia, Erymanthia Achaea, 25015 Greece; 12 Global Brain Health Institute, School of Medicine, Trinity College Dublin, The University of Dublin, Lloyd Building Trinity College Dublin, Dublin 2, Dublin, Republic of Ireland; 13 Centre for Health Services Research, Faculty of Medicine, The University of Queensland, Level 2, Building 33, Princess Alexandra Hospital campus, Woolloongabba, QLD 4102 Australia; 14 Cognition, and Psychotherapy Lab, Department of Psychiatry, Weill Cornell Institute of Geriatric Psychiatry, Weill Cornell Medicine, 315 East 62nd Street, 5th Floor, New York, NY 10065 USA; 15 Department of Informatics and Telecommunications, National and Kapodistrian University of Athens, Panepistimiopolis, Athens, Ilissia 15784 Greece; 16 Department of Psychiatry and Psychotherapy, Klinikum rechts der Isar, Technical University of Munich, Ismaninger Str. 22, 81675 Munich, Germany

**Keywords:** Telemedicine, Dementia, Mild cognitive impairment, Old-age depression, Remote areas

## Abstract

**Background:**

Even though communities in low-resource areas across the globe are aging, older adult mental and cognitive health services remain mainly embedded in tertiary- or secondary hospital settings, and thus not easily accessible by older adults living in such communities. Here, the iterative development of INTegRated InterveNtion of pSychogerIatric Care (INTRINSIC) services addressing the mental and cognitive healthcare needs of older adults residing in low-resource areas of Greece is depicted.

**Methods:**

INTRINSIC was developed and piloted in three iterative phases: (i) INTRINSIC initial version conceptualization; (ii) A 5-year field testing in Andros island; and (iii) Extending the services. The INTRINSIC initial version relied on a digital platform enabling videoconferencing, a flexible battery of diagnostic tools, pharmacological treatment and psychosocial support and the active involvement of local communities in service shaping.

**Results:**

Ιn 61% of the 119 participants of the pilot study, new diagnoses of mental and/or neurocognitive disorders were established. INTRINSIC resulted in a significant reduction in the distance travelled and time spent to visit mental and cognitive healthcare services. Participation was prematurely terminated due to dissatisfaction, lack of interest or insight in 13 cases (11%). Based on feedback and gained experiences, a new digital platform, facilitating e-training of healthcare professionals and public awareness raising, and a risk factor surveillance system were created, while INTRINSIC services were extended to incorporate a standardized sensory assessment and the modified problem adaptation therapy.

**Conclusion:**

The INTRINSIC model may be a pragmatic strategy to improve access of older adults with mental and cognitive disorders living in low-resource areas to healthcare services.

## Background

Designing sustainable services for older adults with mental and cognitive health problems who live in low-resource settings like remote or secluded areas embodies a major challenge for health care systems [1]. As the population worldwide grows and ages, the number of adults affected by age-related brain disorders is expected to increase significantly in the next decades [2, 3]. Of note, the population age distribution is strongly related to the accessibility of a place, and in remote areas, aging populations may represent up to 60% of the overall population [4]. Remoteness and insularity undermine access to basic healthcare and other services [4–7]. There is an urgent need to design and develop pragmatic, feasible and sustainable healthcare services for older adults with cognitive and mental disorders living in low-resource settings.

Old-age mental and cognitive health services are tailored to the specific needs of older people. In such services, even mild forms of aging-related, cognitive and mental disorders can be recognized, and appropriate management is provided [8]. In addition, short psychotherapeutic interventions may also embody an arrow in the therapeutic quiver [9, 10], as is a consideration of common co-morbidities such as the impact of age-related sensory loss (e.g. presbycusis and vision loss) on cognitive function, affective symptoms and functional performance [11, 12]. Nonetheless, such services are in most cases available exclusively in secondary or tertiary healthcare settings and they are not easily accessible by older adults living in low-resource areas [8]. Lack of access to trained clinicians and cost embody important barriers to diagnosis and care according to a report by Alzheimer’s Disease International [13].

Primary healthcare may play a pivotal role in addressing old-age mental and cognitive health needs in low-resource settings, like remote areas [14]. In such contexts primary healthcare providers have de facto a broad role in the diagnosis and management of aging-related, cognitive and mental disorders [15]. A few pioneering, collaborative initiatives have paved the way towards partnerships aiming to improve dementia services for people living low-resource areas [14, 16]. For instance, in Norway more than 90% of the municipalities have local authority dementia teams, consisting of registered nurses and occupational therapists, who closely collaborate with general practitioners in dementia diagnosis and management [16]. In addition, founded on team-based care, decision support, and specialist-to-provider support, the Rural Dementia Action Research Team (RaDAR) primary healthcare model for dementia, being a tertiary-, primary healthcare partnership, provides services to a Canadian community of 1000 people [14]. The model primarily relies on the adaptation of an existing decision support tool for dementia diagnosis and management, the integration of team-based care coordination into the decision support tool, family-centered case conferences, and educational sessions [14]. Interestingly, the coronavirus disease 2019 (COVID-19) pandemic crisis has shed light on the potential utility of pragmatic old-age mental telehealth services [17–21]. Thus, telepsychiatry may prove a chief asset in catalyzing the process toward tertiary-, primary healthcare partnerships in order to meet the mental healthcare needs of older adults residing in low-resource settings like remote areas.

The INTegRated InterveNtion of pSychogerIatric Care (INTRINSIC) services are collaborative mental telehealth services at primary healthcare centers in low-resource areas in Greece. Based on a digital platform, INTRINSIC aims are two-fold: (i) to provide training, support and service development opportunities for primary healthcare professionals serving in hard to access areas of Greece and (ii) to facilitate early detection, monitoring and management of symptoms of adults with age-related brain disease residing in such areas. Here, we describe the conceptualization of INTRINSIC services, their iterative development in a real-world setting, feasibility and acceptability data derived from the pilot study and the final version of the services which now operate on a national level.

## Methods

This was a pragmatic service development and evaluation program. INTRINSIC services were developed and piloted in the three iterative phases: Phase 1: Developing the pillars of the initial version of INTRINSIC; Phase 2: Field testing of the initial INTRINSIC version; and Phase 3: Extending the services, based on feedback and gained experiences.

### Conceptualization of INTRINSIC services (phase 1)

INTRINSIC services were initially based on the following four pillars:

### The digital platform

INTRINSIC digital platform was based on the prototype ‘Synergasia’ (https://synergasia.uoa.gr/). The platform enabled synchronous communication between professionals of the university old-age psychiatry unit and the primary healthcare center and the assessment of patients at their home or at the primary healthcare center by old-age psychiatry professionals via videoconferencing. The platform provided digital services that work intuitively through easy-to-use user interfaces and that worked equally well on all devices (laptops, tablets, smartphones). In most cases laptops were used. The communication between the user’s browser and the service was protected with the standard https protocol, with a valid certificate, so it was safe from eavesdropping by others on the same network. Regarding the video conference set-up, the simplest solution was selected, given that the participating health center lacked any advanced videoconference equipment. Each end used a simple laptop computer, using the built-in webcam and microphone. The videoconference solution was tested to provide an adequate video and audio quality, provided the local network connection at each end meets our recommendations. We expected that the camera at each end will be positioned by the user in the best way possible given the aforementioned set-up. Online consultations for which the service user went to the primary healthcare center in Andros were rarely hampered by poor network connection. In case of online consultations in settings with no available internet connection, i.e. home visits, Wi-Fi connection by tethering was shared.

*A flexible battery of tools for assessment and monitoring of older adult mental and cognitive health*.

Age-related brain disease symptoms were assessed with a flexible battery of neurocognitive tools [22], including the Mini-mental State examination (MMSE) [23],the Montreal Cognitive Assessment (MOCA) [24], the MiniCog [25], the Frontal Assessment Battery (FAB) [26] and the Geriatric depression scale − 15 (GDS) [27]. Based on the findings of the neurocognitive assessment and on a psychiatric assessment via videoconferencing the clinical diagnosis was established according to the DSM-5 diagnostic criteria [28]. During online consultations a primary healthcare professional sat next to INTRINSIC service user to handle technical issues that arose and facilitate communication between the psychiatrist and the service user. Follow-up assessments took place at home or at the primary healthcare center either by local primary healthcare professionals or by both local healthcare professionals and an old-age psychiatry professional, who contributed to the assessment via videoconferencing.

### Pharmacological treatment and psychosocial support of older adult mental and cognitive health

The therapeutic endeavors included both pharmacological treatment and psychosocial support. The pharmacological treatment plan was developed by professionals of the old-age psychiatry unit, while the psychosocial interventions, which were delivered by primary healthcare professionals, were pragmatically restricted to individualized patient- and care partner counseling. It primarily comprised psychoeducative elements, assessment of care needs, referrals to community welfare services and the development of coping strategies tailored to the needs of the person with old-age mental disorder and his/her care partner [29].

### Active local community involvement

Patients, care partners, families and active members of the local communities were involved in the development of INTRINSIC services.

### Field testing of the initial version of INTRINSIC (phase 2)

#### Design

To field test INSTRINSIC, we undertook a pragmatic, observational, service development and evaluation approach, based in real-life clinical settings and scenarios.

### Setting

Coordinated by the old-age psychiatry unit of the Eginition University Hospital in Athens, the INTRINSIC pilot study took place between 2015 and 2020 in the Andros Island. Andros is the second largest island of the Cyclades islandic group in the Aegean Sea. It is characterized by many remote villages that are scattered throughout the island hampering access to specialized old-age psychiatry services. Andros has a permanent population of approximately 2,203 older adults. All older adults permanently residing in Andros were informed about the INTRINSIC services by word-of-mouth or referred to them by their general practitioners or primary healthcare professionals, while a public outreach event was organized once per year in different areas of the island to raise awareness about the services. Of note, organizations of the civil society, i.e. local clubs and the local Christian-Orthodox Church, spread the word about the services.

### Service users

Inclusion criteria included (i) interest in using INTRINSIC services, (ii) age 60 and higher (iii) being an inhabitant of Andros. Exclusion criteria included (i) no permanent residence in Andros (ii) age younger than 60, (iii) no subjective complaints and no family concerns related to symptoms of neurocognitive disorders and/or further mental disorders.

### Evaluation framework

To ascertain whether the first version of INTRINSIC would be feasible and have utility, we examined the following outcomes: (1) service user profile, i.e. mental and/or neurocognitive diseases of participants, and number of new diagnoses; (2) description of service use (performed diagnostic tests, number of online consultations, number of consultations at home and at the primary healthcare center); (3) service user attrition, i.e. number or premature termination of participation; (4) changes in barriers to cognitive- and mental healthcare (distances travelled and time spent for travelling and accessing healthcare services due to old-age mental or neurocognitive disorder and returning home); (5) INTRINSIC stakeholder feedback (service users, care partners, professionals).

### Ethical considerations

The INTRINSIC protocol and its implementation conform to the principles of the sixth revision of the Declaration of Helsinki and was approved by the Institutional Review Boards of the involved healthcare centers. Written informed consent was obtained from all pilot phase INTRINSIC service users or authorized representatives prior their enrollment.

### Statistical analyses

Normal distribution of INTRINSIC data was checked using the Kolmogorov-Smirnov test. Statistical comparisons of annual mobility data prior and posterior to enrollment in INTRINSIC were performed with the Wilcoxon Signed Ranks Test. A p value of < 0.05 was considered to indicate statistical significance. Statistical analyses were implemented in IBM SPSS Statistics 27 for Windows.

## Results

### Service user profile and % of new diagnoses

The INTRINSIC pilot phase services were taken up by 119 people (0.05% of older adults living in Andros). Their demographic and clinical characteristics are presented in Table 1. In most service users, neurocognitive disorders and/or depressive disorders were diagnosed. Of note, participation in the pilot study resulted in newly diagnosed mental and/or neurocognitive disorders in 72 service users (61%).

**Table 1:** Demographic and clinical characteristics of users of integrated intervention of psychogeriatric care services at Andros Island between 2015–2020 (pilot phase)

Characteristics	
N	119
Age, years*	75.2 (9.45) [60–97]
Education, years*	7.29 (3.28) [1–16]
Gender (female)	72
Mini Mental State Examination (MMSE)*	N = 63; 23.41 (5.93) [6–30]
Montreal Cognitive Assessment (MOCA)*	N = 6; 18.17 (3.43) [14–23]
MiniCog*	N = 19; 2.68 (1.7) [0–5]
Frontal Assessment Battery (FAB)*	N = 17; 8.82 (4.55) [0–16]
Geriatric Depression Scale 15 (GDS)*	N = 14; 8.07 (4.48) [0–16]
*Mental disorders* ^*≠*^	
Neurocognitive Disorders (N) [diagnosed prior enrollment, N]	46 [17]
Depressive Disorders (N) [diagnosed prior enrollment, N]	39 [19]
Anxiety Disorders (N) [diagnosed prior enrollment, N]	27 [4]
Schizophrenia Spectrum and Other Psychotic Disorders (N) [diagnosed prior enrollment, N]	4 [4]
*Somatic diseases*	
Hypertension (%)	68
Dyslipidemia (%)	33.8
Coronary artery disease (%)	0.03
Heart Failure (%)	0.08
Atrial fibrillation / Arrhythmia (%)	25
Diabetes mellitus (%)	20.3
Stroke (%)	0.06
Chronic Obstructive Pulmonary Disease, Asthma (%)	0.09
Sensory Deficits (%)	46.1
Thyroidopathy (%)	16.4
Neurological Disorders, other than dementia (%)	0.08
Gastrontestinal diseases (%)	12.7
Chronic pain syndrome (%)	26.2
Sum of somatic diseases*	3.22 (1.89) [0–10]

*Mean (standard deviation) [minimum-maximum]

^*≠*^ In nine-, eight- and one INTRINSIC service users both depressive and anxiety disorders, both neurocognitive and depressive disorders and both neurocognitive and anxiety disorders were diagnosed, respectively

### Description of service use

At baseline, 63, 6, 19, 17 and 14 users were assessed with the MMSE, the MOCA, the Mini Cog, the FAB and the GDS, respectively. In total, 732 online consultations, 120 offline consultations at home and 738 offline consultations at the primary healthcare center took place.

### Service user attrition

In the whole group of pilot phase INTRINSIC service users, participation was prematurely terminated due to relocation in nine cases (8%), because of dissatisfaction with services/lack of interest in seven cases (6%), due to death in six cases (5%), because of lack of insight in six cases (5%) and due to organizational and communication difficulties caused by cognitive deficits of the user and lack of a care partner in two cases (2%).

### Changes in barriers to old-age cognitive- and mental healthcare services

**Fig. 1:**
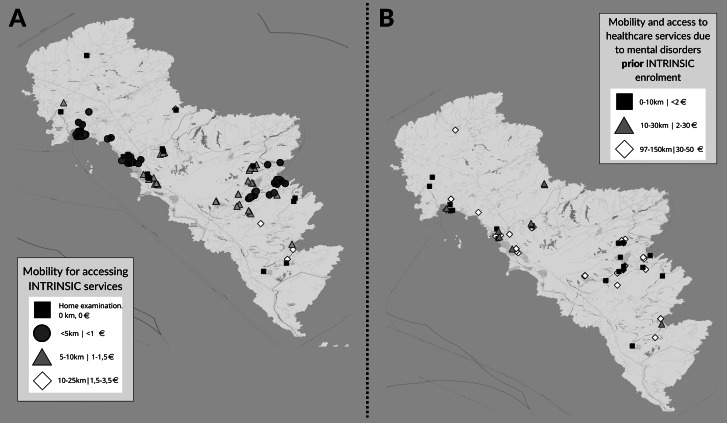
Mobility patterns of participants of the pilot study of INTegRated InterveNtion of pSychogerIatric Care (INTRINSIC) for seeking old-age mental healthcare in the year prior and posterior their enrollment

The use of INTRINSIC services reduced barriers to old-age mental healthcare, since it contributed to the reduction of time and costs for seeking old-age cognitive-and mental healthcare. It resulted in a significant reduction in the distance (in km) older adults annually travelled to visit health care services (mean [standard deviation] prior vs. posterior enrollment: 132.70 [114,20] vs. 6.78 [8.95], Z= -5.99, P < 0.001) and in time (hours) annually spent for travelling and accessing healthcare services due to their old-age mental or neurocognitive disorder and returning home (mean [standard deviation] prior vs. posterior enrollment: 206.41 [175.37] vs. 11.37 [13.80], Z= -5.78, P < 0.001), while the annual number of visits significantly increased (mean [standard deviation] prior vs. posterior enrollment: 1.69 [1.00] vs. 8.71 [5.98], Z= -5.91, P < 0.001). In 22 service users the observation period posterior to enrollment was shorter than twelve months, because they had been enrolled in the last year of the INTRINSIC pilot phase. As expected, the cost of each visit to services significantly declined (mean [standard deviation] prior vs. posterior enrollment: 36.93 [33.88] vs. 1.05 [1.39], Z= -5.97, P < 0.001). The changes in mobility patterns, while seeking healthcare due to cognitive and mental disorders are graphically presented in Fig. 1.

### Stakeholders’ feedback

Feedback was received by all service users, their care partners and involved healthcare professionals. It was unstructured and was requested after each consultation through direct questions to service users and their care partners about how they received the services. Feedback was given in an ad hoc way without constraints or limits. Issues which were raised by service users and their families were the increase of the frequency and/or the duration of offline and/or online consultations, accessibility difficulties of users of INTRINSIC services with severe dementia and or mobility difficulties, and the enrichment of non-pharmacological interventions to treat depression and non-cognitive symptoms of neurocognitive disorders. Feedback from professionals was mainly given during team meetings. Professionals of the primary healthcare center in Andros complained about lack of confidence regarding the management of issues of old-age cognitive and mental health issues at the beginning of the pilot phase, while they increasingly expressed their aspiration to be more actively involved in the development of the individual therapeutic plans after completing the necessary training. Moreover, they expressed criticism regarding the lack of structured training and assessment of cognitive and mental health and complained about increasing workload. They also criticized the absence of assessment of age-related symptoms/constellations (e.g. sensory loss, polypharmacy, falls) that may mirror or interact with brain diseases and may perplex the management of cognitive and mental health of older adults. Moral support and donations of local clubs, the local Christian Orthodox Church, local businesses, and a few service users were sources of positive feedback and encouragement.

### Program extension based on feedback: from the pilot phase to the extended INTRINSIC services (phase 3)

Part way through the Phase 2 field trial, informal feedback from professionals revealed that the digital platform needed to be upgraded to facilitate among others e-training of healthcare professionals, while the diagnostic and therapeutic arms of INSTRINSIC needed to be extended to incorporate three additional components: (1) an old-age cognitive, behavioral and mental health risk factor surveillance system, (2) an additional set of assessments to address sensory health in the context of aging-related cognitive decline and (3) a non-pharmacological therapeutic component, which took the form of a psychotherapeutic intervention, based on modified problem adaptation therapy (M-PATH).

#### These are described below

**Fig. 2:**
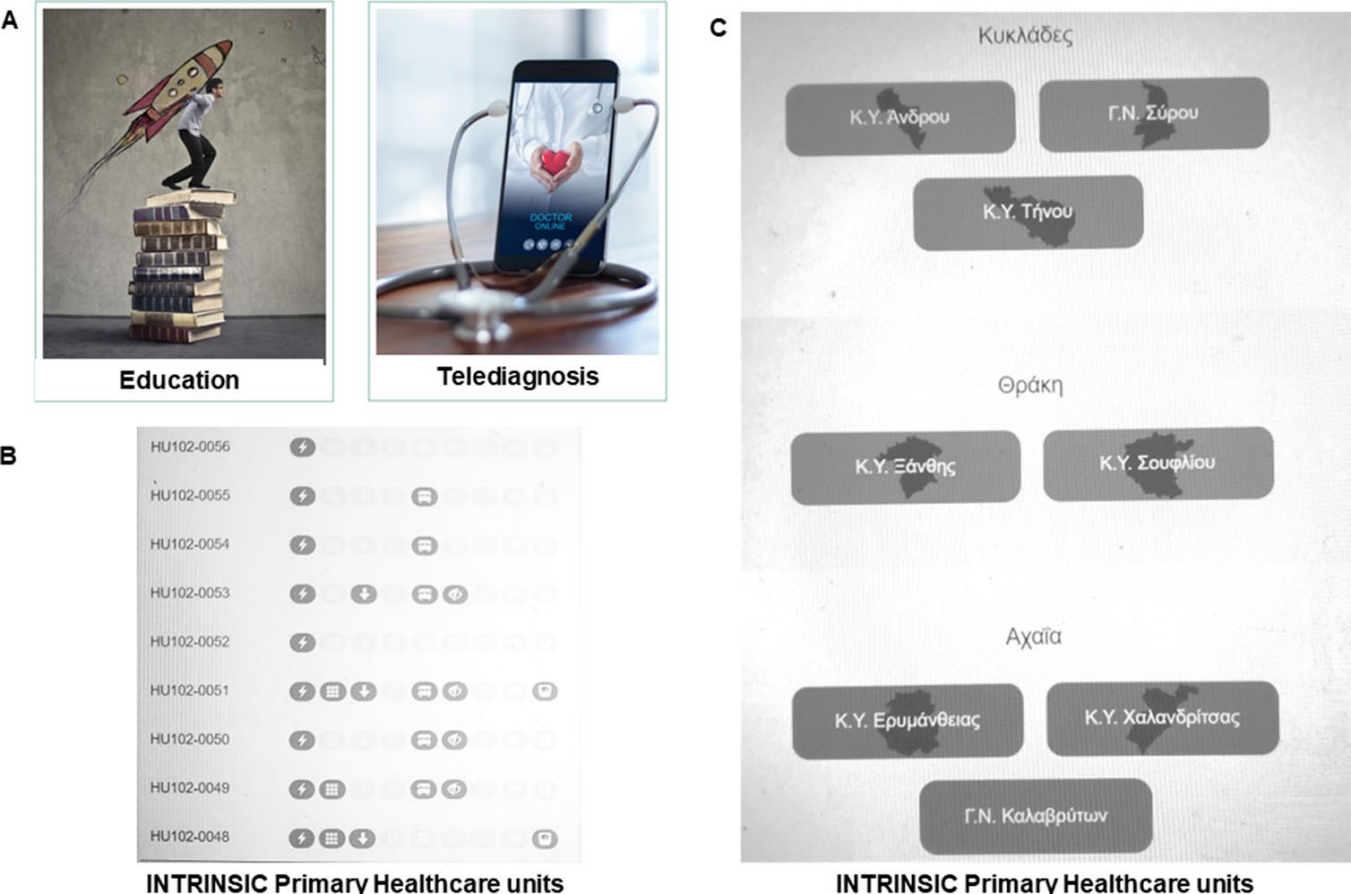
Screenshots of the HEllenic Remote MEntal health Services for old-age (HERMES) digital platform (https://hermes-tk.med.uoa.gr/). **A**: The platform integrates a system of telediagnosis, the surveillance system of old-age mental health risk factors, an e-training system for professionals of the INTegRated InterveNtion of pSychogerIatric Care (INTRINSIC) and an open to the public digital library with information and awareness raising materials about mental health in aging; **B**: Red flags raised after completion of clinical data of nine users of INTRINSIC services in the surveillance system; **C**: Members of the INTRINSIC network have access and fill their regional database of the surveillance system

The HEllenic Remote MEntal health Services for old-age (HERMES) digital platform (https://hermes-tk.med.uoa.gr/) is the upgraded INTRINSIC digital platform. It integrates: (i) an innovative system of telediagnosis and surveillance of old-age cognitive and mental risk factors, (ii) an e-training system for INTRINSIC professionals which offers self-paced learning courses and webinars in state-of-the art psychogeriatric topics and (iii) an open to the public digital library with awareness raising materials about mental health in aging (Fig. 2). The platform enables synchronous and asynchronous communication between the involved professionals, as well as INTRINSIC participant assessment and counseling. The videoconferencing is served via separate dedicated server only used for the HERMES platform, and video and audio is end-to-end encrypted between the participating users. Access control is enforced using the accounts from the Open eClass platform, and each user present in the videoconference room is prominently displayed in the list of participants.

The old-age cognitive, behavioral and mental health risk factor surveillance system is a premier system of detection of signs or risk factors that constitute alarm bells (red flags) for the mental and cognitive health status of service users. Building on the Behavioral Risk Factor Surveillance System of the Centers for Disease Control and Prevention [30], it relies on a 20-item questionnaire enabling the recording of nine distinct warning constellations, i.e. polypharmacy, falls, weight loss, insomnia, cognitive complaints, traumatic/stressful events, depressive symptoms, anxiety symptoms and cognitive impairment. The questionnaire is administered by healthcare professionals at the primary healthcare centers after the necessary training by old-age psychiatrists involved in the INTRINSIC services. The surveillance system comprises items of the instruments General anxiety Disorder 2-item (GAD-2) [31], Patient Health Questionnaire-2 (PHQ-2) [32], and Mini-Cog, while the presence of cognitive complaints is assessed with questions about increasing memory difficulties, difficulties to remember recent events, names and/or recently memorized information, naming difficulties, difficulties with misplacing objects and/or feeling “foggy”. The system is hosted on a dedicated virtual server based on the proven Open eClass platform. The data are anonymously stored locally in a database on the same server and the red flags are computed dynamically during data display from the stored data of each person’s record, based on appropriate thresholds for each flag. Red flags lead to extensive diagnostic workup related to them and to appropriate personalized pharmacological and psychosocial interventions at the primary healthcare center or at home. In addition, they generate a region-specific map of mental and cognitive health problems and risk factors, which can form a solid empirical basis for developing tailored primary, secondary and tertiary prevention strategies in low-resource areas.

The sensory assessment set arose from an EU funded research program investigating the interplay of hearing, vision and cognitive decline to improve outcomes in older adults (https://www.sense-cog.eu) [12]. It aims to contribute to early diagnosis and personalized care of people suffering from both age-related sensory loss and cognitive deficits. INTRINSIC service users are assessed for possible hearing and vision impairment by primary healthcare professionals who are trained and supervised by a sensory therapist based at the Eginition University Hospital.

The Modified Problem Adaptation Therapy (M-PATH) enriched the therapeutic quiver of INTRINSIC services [33]. M-PATH provides psychotherapeutic treatment in older adults with depression with or without minor neurocognitive disorder and their care partners. It aims to shift the balance of negative-positive emotions in favor of positive ones as well as to facilitate problem solving and adaptive functioning. The training of primary healthcare professionals involved in INTRINSIC in the theoretical background of M-PATH consists of two introductory six-hour workshops and two further three-hour workshops focusing on M-PATH clinical implementation and on trainees’ empowerment with the PATH approach. The M-PATH implementation at primary healthcare centers includes scheduled psychotherapeutic sessions taking place at the centers and monthly videoconferencing-based supervision sessions, provided by M-PATH experts.

**Fig. 3:**
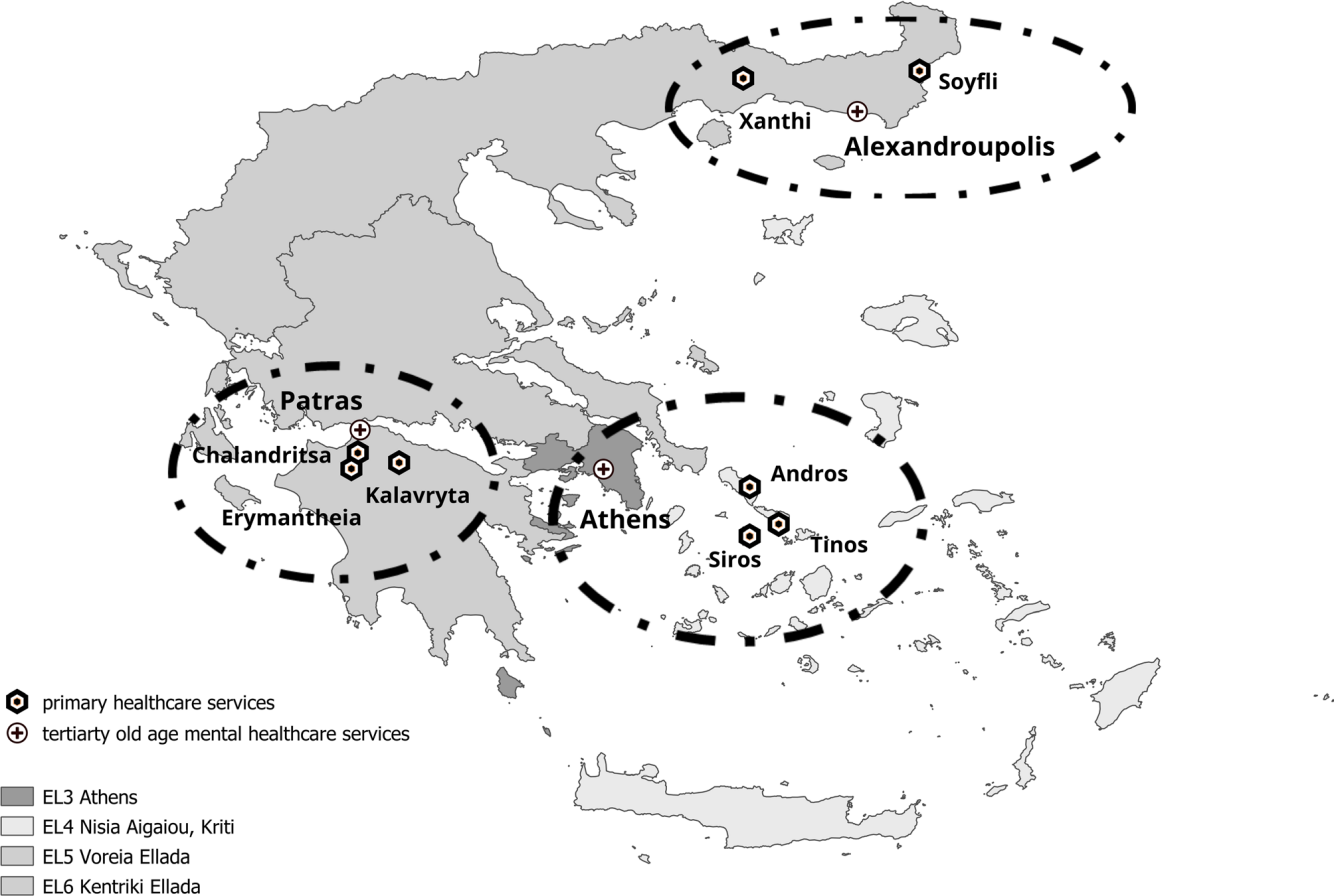
Old-age psychiatry university units- and primary healthcare services involved in INTegRated InterveNtion of pSychogerIatric Care (INTRINSIC)

The extended INTRINSIC services are offered since May 2022 by eight primary healthcare centers in four different Nomenclature of Territorial Units for Statistics (NUTS) 1 regions of Greece in cooperation with three tertiary psychogeriatric units (Fig. 3) and is funded by the Greek Ministry of Health and Social Solidarity.

## Discussion

INTRINSIC services embody a novel and service user-friendly way to reduce barriers to old-age cognitive and mental healthcare services of older adults living in low-resource areas through taking advantage of telehealth. They rely on the amplification of intrinsic resources and skills of network partners. INTRINSIC has a four-dimensional crossover design: (i) it links already operating tertiary health care services with primary health care; (ii) it overcomes the deeply embedded institutionalized dualism in health care services (health services focusing either on physical or on mental health) [34]; (iii) it is based on collaborating medical and non-medical healthcare professionals; (iv) it comprises both pharmacological and pragmatic nonpharmacological therapeutic interventions. Of note, the increasing digital literacy of both older adults and healthcare professionals was a key aspect in the conceptualization and successful implementation of INTRINSIC services [20, 35]. The user-friendliness of INTRINSIC services may be reflected in the relatively low attrition observed in the pilot study. Moreover, the reduction in the barriers to old-age cognitive and mental health services is reflected in the significant decrease in the distance annually travelled and in time annually spent for accessing healthcare services due to age-related brain disorders, while the cost of each visit to services significantly declined. Indeed, attrition in INTRINSIC services was similar to that observed in a large study of older adults who were treated with psychological therapies in the United Kingdom, for the treatment effectiveness of which clients’ motivation and satisfaction are key aspects [36–38]. In addition, attrition in INTRINSIC services was lower compared to the treatment dropout rates in people from 13 low- or middle-income countries and 15 high-income countries who suffered from mental and substance use disorders, which was found to exceed 30% and did not pertain to socio-demographic variables (e.g. age) [39].

INTRINSIC services may be a pragmatic model to address inequity in mental and cognitive health care of older adults living in low-resource areas. Considering the fact that people living with dementia in low-resource settings are less likely to have a diagnosis [40, 41] and that the primarily somatic character of depressive symptoms in older adults perplexes their detection [42], it is hardly unexpected that in 61% of INTRINSIC pilot phase service user new diagnoses of neurocognitive and/or mental disorders were established. However, it cannot be excluded that the high frequency of neurocognitive and/or mental disorders is attributed to the relatively low uptake of services by the older adults residing in Andros (119/2,203). Nevertheless, the detected frequencies of cognitive impairment and depressive disorders in INTRINSIC users in Andros island lay between frequencies reported in epidemiological studies and studies conducted in primary healthcare settings in Western World rural areas [43, 44]. INTRINSIC is a model of integrating cognitive and mental healthcare services into primary healthcare, the professionals of which are the key actors in managing health issues of older adults in low-resource communities. Interestingly, cognitive and/or mental healthcare services provided in primary healthcare bypass the dominant institutionalized dualism, separating mental- from physical healthcare services, which may contribute to stigma against mental illness [45, 46]. The INTRINSIC old-age cognitive, behavioral and mental risk factor surveillance system and sensory health assessment enable a pragmatic strategy to detect warning constellations related to brain health, such as falls, sleep disturbances and polypharmacy [47–50], without overwhelming older adults with exclusive and extensive focus on mental disorder symptoms. In addition, cognitive and/or mental healthcare is delivered at primary healthcare centers by their staff, not including mental health professionals. In such a way fears of stigmatization and low levels of psychological openness, which are particularly prevalent in low-resource communities [51, 52], may be overcome. The relatively low number of participants who discontinued using INTRINSIC services due to dissatisfaction with services/lack of interest and lack of insight in the pilot phase may point to the efficiency of services in bypassing stigma around mental illness in low-resource communities.

Patients, care partners and local communities have a shaping role in INTRINSIC. The encouraging and supportive attitude of the members of the local INTRINSIC advocacy body and organizations of the civil society in Andros was a source of motivation for all INTRINSIC professionals. Their suggestions, ideas, comments and moral and financial support have contributed to the further development of the design of INTRINSIC as well as to building trust with the local communities. For instance, the structured e-training system for INTRINSIC professionals was developed based on the educational needs of primary healthcare professionals in Andros regarding old-age cognitive and/or mental health issues. Furthermore, the enrichment of INTRINSIC with a pragmatic psychotherapeutic component was advocated by service users. In line with previous reports, their support for non-pharmacological management of aging-related morbidities may be seen as a way to foster self-determination and continued opportunities for meaning and purpose for older adults with brain diseases and their care partners [53]. It is likely that non-pharmacological treatment relies on a more active involvement of patients and their care partners in the management of late-onset depressive symptoms or symptoms of neurocognitive disorders compared to being exclusively on medication [54].

The pilot phase of INTRINSIC services unveiled factors that may complicate the implementation process of such services. Lack of confidence of primary healthcare professionals in managing issues of old-age cognitive and mental health, accessibility difficulties and increasing workload were the issues that were mainly raised by primary healthcare professionals in team meetings when encountered obstacles were discussed. Training materials tailored to the needs of primary healthcare professionals were developed after the pilot phases and prior to the extension of INTRINSIC services and contributed to building in the primary health professionals of the extended network of INTRINSIC services of a solid foundation of knowledge and understanding of old-age cognitive and mental healthcare issues, while consensus meetings via videoconferencing build confidence regarding diagnostic and therapeutic issues and deepen knowledge [55]. For users of INTRINSIC services with severe dementia and/or mobility difficulties home visits were a pragmatic solution. Home visits and positive response to requests for an increase in frequency of offline and/or online consultations in several cases increased workload. Time management, work regulation and peer support are effective strategies in reducing occupational stress [56, 57]. In addition, the inclusion of healthcare professionals with values like commitment to equity in access to healthcare services, empathy, compassion, and openness into INTRINSIC has also fueled the efforts to overcome the aforementioned obstacles in service implementation.

The INTRINSIC pilot study has a number of limitations. First, 22 older adults began to use INTRINSIC services in the last year of the pilot phase, so that the collected data regarding the distance they travelled and the time they spent for seeking mental healthcare services after their enrollment refer to a period of less than twelve months and may have biased our findings related to the detected significant decrease in these parameters after pilot study enrollment. Nonetheless, the impact of these cases on our findings may be relatively limited taken into account that the number of visits to mental healthcare services significantly increased in the period after enrollment compared to the year that preceded enrollment. Furthermore, the old-age cognitive, behavioral and mental risk factor surveillance system, the M-PATH and sensory assessment and support components of INTRINSIC became available to users only after the end of the pilot phase. Thus, pilot phase data refer only to a part of the current, extended INTRINSIC services. Nevertheless, here a pragmatic service development and evaluation program is depicted. No data on the cost of INTRINSIC services or on the impact of the impact of the environment of online consultations on the interaction between healthcare professional and service user [58] were collected in the pilot phase since it was a phase of iterative development of the services. A further shortcoming of the study stems from the implementation of the INTRINSIC pilot phase in an island. Insular low-resource areas differ compared to that of low-resource areas in mainland territories [59, 60]. Hence, our results may not be generalizable to non-insular low-resource communities. The current INTRINSIC network including healthcare centers in four different NUTS1 regions in Greece, embodies a valuable source of data, which could provide further evidence for the acceptability and feasibility of INTRINSIC services.

## Conclusions

Overall, realistic ways towards equity in old-age cognitive and mental healthcare services particularly in low-resource areas are urgently needed. INTRINSIC is a telehealth model of an integrated intervention of psychogeriatric care based on a network of old-age university- and low-resource primary healthcare units. The one-site pilot study of INTRINSIC provides first evidence of the integrity and acceptability of INTRINSIC services.

## Data Availability

The datasets used and analyzed during the current study are available. from the corresponding author on reasonable request.

## Abbreviations

INTRINSIC: INTegRated InterveNtion of pSychogerIatric Care
COVID-19: Coronavirus disease 2019
MMSE: Mini-mental State examination
MOCA: Montreal Cognitive Assessment [17]
FAB: Frontal Assessment Battery
GDS: Geriatric depression scale − 15
DSM-5: Diagnostic and Statistical Manual of Mental Disorders, Fifth Edition
M-PATH: Modified problem adaptation therapy
HERMES: HEllenic Remote MEntal health Services for old-age
GAD-2: General anxiety Disorder 2-item
PHQ-2: Patient Health Questionnaire-2
EU: European Union
NUTS1: Nomenclature of territorial units for statistics major socio-economic regions
